# Editorial: Advancements in nanomedicine for targeted cancer therapy and imaging

**DOI:** 10.3389/fbioe.2026.1848448

**Published:** 2026-04-28

**Authors:** Nemany A. N. Hanafy, G. Caruso, Stefano Leporatti

**Affiliations:** 1 Koç University Research Center for Translational Medicine (KUTTAM), Istanbul, Türkiye; 2 Group of Bionanotechnology and Molecular Cell Biology, Nanomedicine Department, Institute of Nanoscience and Nanotechnology, Kafrelsheikh University, Kafrelsheikh, Egypt; 3 University Hospital of Policlinico G. Martino, Messina, Italy; 4 CNR NANOTEC-Istituto di Nanotecnologia, Lecce, Italy

**Keywords:** cancer, imaging, nanocarriers, nanomedcine, target therapy

Chemotherapy is extensively employed in cancer clinical therapy, yet prolonged exposure to chemotherapy agents can cause multi-drug resistance, bone-marrow suppression, and other undesirable effects (Zhao et al., 2019). On the other side, tumour heterogeneity, inadequate vascularization, and elevated interstitial pressure frequently prevent therapeutic compounds from attaining a sufficiently high concentration in cancer cells. The conventional chemotherapeutic drugs exacerbate these problems by circulating throughout the body, resulting in greater toxicity and diminished drug efficacy. To improve the survival and cure rate of cancer patients, a revolutionary targeted tumour treatment strategy is proposed (Gao et al., 2018). A targeted drug can effectively promote the uptake of a drug at a tumour site to improve the therapeutic effect. On this prospective, this strategy leads to reduce side effects on non-targeted cells. The nano-platform outlined in the study consists of biodegradable components that are capable of being safely metabolized after releasing the therapeutic agents (Deivayanai et al., 2024). In contrast to conventional nanoparticles that could be retained in non-target tissues, this system seeks to reduce potential hazards arising from prolonged exposure to the body while still being stable enough to travel through the bloodstream until tumor cells are encountered. Importantly, the platform incorporates molecular features that allow for both targeted treatment and imaging. The inclusion of imaging-compatible components enables real-time tracking of nanoparticle distribution, offering insight into tumour uptake and therapeutic response (Hanafy, 2021). This dual functionality could be particularly valuable in assessing treatment effectiveness early during therapy and adjusting clinical strategies accordingly. Since the inaugural use of a monoclonal antibody (mAb) on a lymphoma patient in 1980, antibodies with remarkable specificity have become powerful agents for targeted cancer treatment and precise diagnosis (Warda et al., 2018). In this context, two strategies have recently been developed: passive and active targeting therapies. Passive targeting allows nano-vectors to accumulate in the tumour microenvironment because of distinct characteristics of the tumor that are absent in normal tissues. The nanocarriers can preferentially gather in cancerous tissue through passive targeting owing to the enhanced permeability and retention (EPR) (Kang et al., 2021). Active targeting schemes for nanoparticle systems in cancer therapeutics (Narwade et al., 2023). The EPR derives from key features of tumor tissue: leaky blood vessels, deficient lymphatic drainage, and the nanometer scale of the carriers. Active binding to target cells and clearance by the RES constitute two kinetically opposing processes. Nonetheless, surface receptors or epitopes enable nanocarriers to recognize and attach to target cells via ligand-receptor binding. To overcome the drawbacks of passive targeting, affinity ligands (e.g., small molecules, peptides, antibodies, and aptamers) that selectively engage specific receptors can be grafted onto the carrier surface using diverse conjugation chemistries (Byrne et al., 2008). Because small-molecule ligands readily form linkages with proteins, a single ligand can interact with multiple targets. Using this approach, the creation of nanoscale carrier-conjugated antibodies, specific molecules, and peptides has enhanced the efficacy of cancer therapies, reduced off-target toxicities by increasing drug delivery to intended cells, and improved drug stability by shielding them from degradation. The method also lessens hemolysis of blood cells by limiting nanoparticle-cell interactions. In contrast, direct exposure of blood cells to chemotherapeutics can lead to loss of cells and acute anemia. Active targeting approaches aim for higher accuracy and selective delivery to malignant tissue compared with passive targeting, which depends solely on EPR. Accordingly, nanoparticles may be loaded with one or several agents and delivered to tumors via either active or passive targeting routes (Hanafy and Kolemen, 2026).

In conclusion, these studies and reviews are excellent examples of cutting-edge research and information that have been offered by many international research groups focusing on bioimaging and targeting nanoparticles. The papers included in this special e-collection unequivocally demonstrate that there are still many aspects of drug delivery system synthesis, characterisation, and applications that need to be addressed and understood, despite the abundance of current literature and data connected to this highly important Research Topic.
